# Inferior Vena Cava Draining Into the Left Atrial Cavity Due to Atrial Septal Defect: Two Atypical Presentations

**DOI:** 10.7759/cureus.39159

**Published:** 2023-05-17

**Authors:** Avinash Prakash, Pratik Pandey, Isha P Khonde, Ayesha Goyal

**Affiliations:** 1 Cardiothoracic Surgery, Sri Sathya Sai Sanjeevani Hospital, Raipur, IND; 2 Cardiac Anaesthesia, Sri Sathya Sai Sanjeevani Hospital, Raipur, IND

**Keywords:** septum secundum, interatrial septum, left atrium, inferior vena cava, atrial septal defect

## Abstract

The inferior vena cava draining to the left atrium is a rare congenital anomaly. Patients usually present with hypoxia and dyspnoea. This condition is usually diagnosed by echocardiography and sometimes by CT scan. Here we report two cases that presented with normal saturation and their surgical management.

## Introduction

It is very rare for the inferior vena cava (IVC) to empty into the left atrium (LA) when the normal atrial configuration (situs solitus) is present [1]. It can occur with atrial septal defect (ASD), anomalous pulmonary venous drainage, and pulmonary arteriovenous fistula [2]. In the literature, several cases with various anatomical associations have been published [3-9]. We outline two cases of patients who exhibited this specific abnormality and how it was treated.

## Case presentation

**Figure 1:**
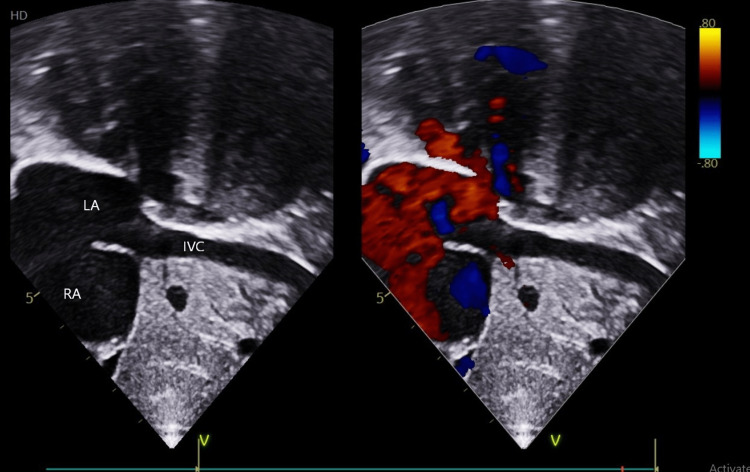
Case 1 - Echocardiography showing IVC draining into the LA. IVC: inferior vena cava; A: left atrium; RA: right atrium

The first case is a six-year-old male child who was incidentally diagnosed with ostium secundum atrial septal defect (OSASD). Physical examination revealed a resting saturation of 99% in room air and a systolic murmur. His electrocardiogram (ECG) and chest radiograph were normal. A transthoracic echocardiogram revealed a 17 x 20mm OSASD with IVC draining into LA (Figure 1).

**Figure 2:**
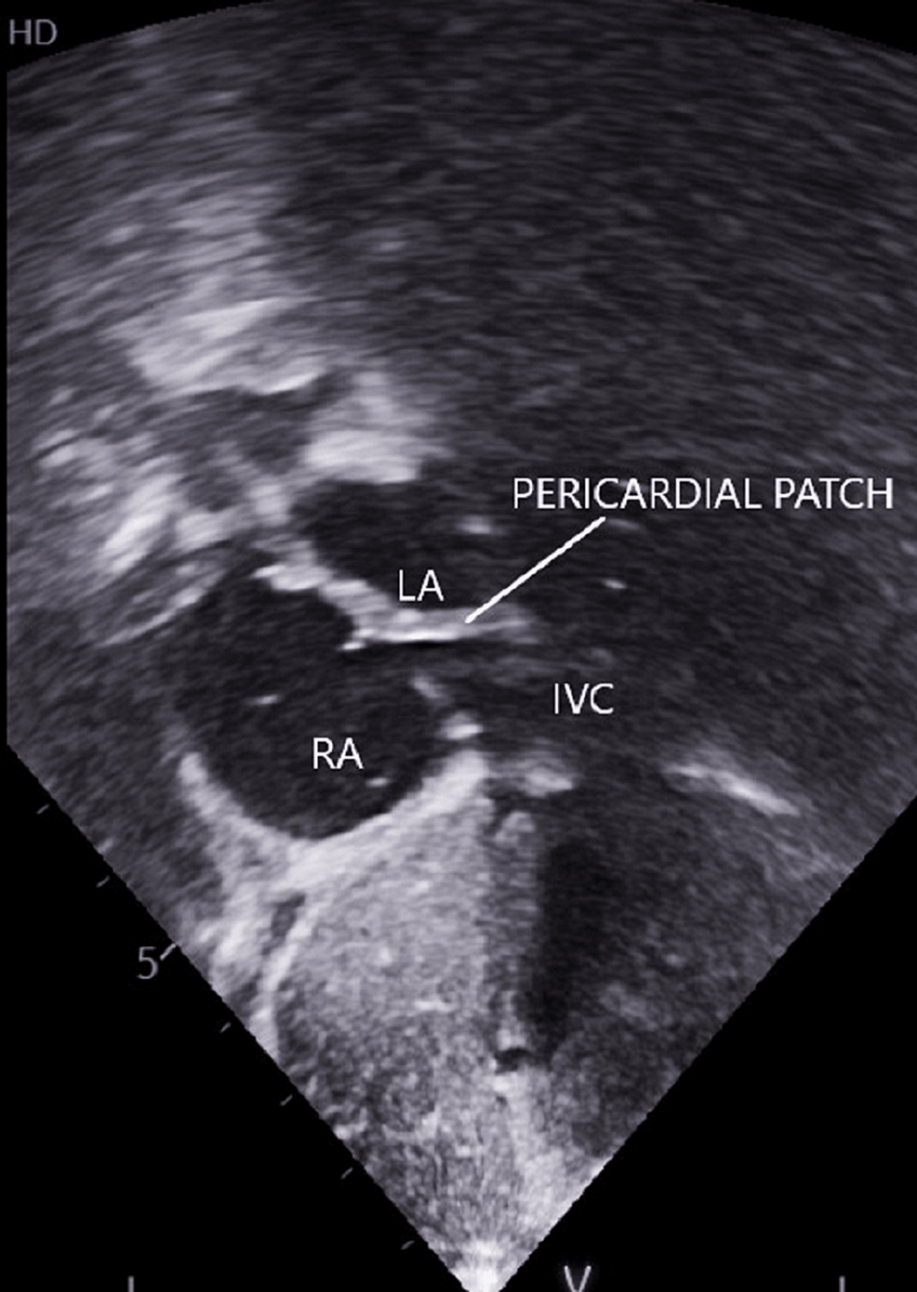
Case 1 - Postoperative echocardiography showing the oblique orientation of the pericardial patch. LA: left atrium; RA: right atrium, IVC: inferior vena cava

After a proper preoperative check-up, he was scheduled for OSASD closure. Intraoperatively, it was observed that the IVC opening was posterior to the septal margin. The IVC was then baffled into the right atrium (RA) using an oblique autologous pericardial patch. The patient tolerated the procedure well and was discharged a few days later, asymptomatic and with normal oxygen saturation. Echocardiography on a one-month follow-up showed unobstructed SVC and IVC draining into RA (Figure 2).

**Figure 3:**
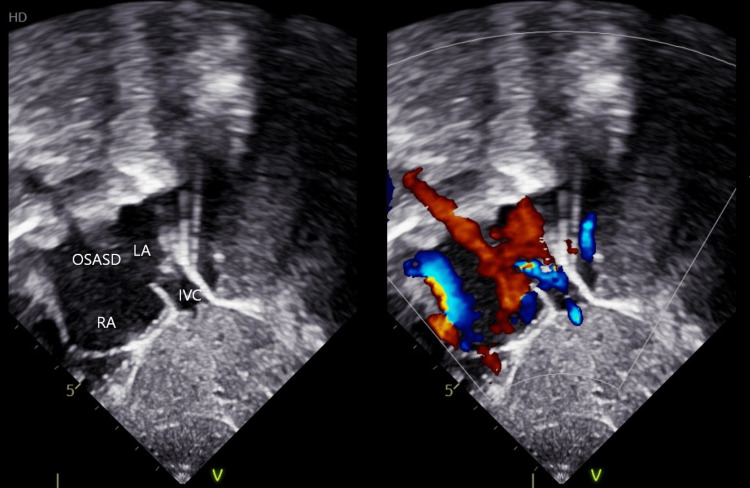
Case 2 - Echocardiography showing IVC draining into the LA. IVC: inferior vena cava: LA: left atrium, RA: right atrium

The second case involves a four-year-old female child who was also incidentally diagnosed with ostium secundum atrial septal defect (OSASD). Physical examination revealed a resting saturation of 99% in room air and a systolic murmur. ECG and chest radiographs were normal. A transthoracic echocardiogram revealed a 20 mm posterior OSASD with IVC draining into LA (Figure 3).

Left-sided superior vena cava draining into the coronary sinus was also noted. Intraoperatively all margins of the interatrial septum were well formed. It was noticed that the IVC was opening into the LA and IAS was malaligned. The IVC was then baffled into the RA using an oblique autologous pericardial patch. The patient tolerated the procedure well and was discharged three days later, asymptomatic and with normal oxygen saturation. Echocardiography taken at the one-month follow-up showed unobstructed SVC and IVC draining into RA.

## Discussion

Gardner originally identified the unusual congenital vascular condition known as abnormal IVC draining into the left atrium in 1955 [10]. Most patients with anomalous venous return present early with signs of right to left shunt-like hypoxia, dyspnea, cyanosis, murmur, and clubbing [11]. The systemic sinus venosus and the primary part of the RA are separated during the early stages of embryologic development by the right and left venous valves [12,13]. The crista terminalis, Eustachian, and Thebesian valves are typically all that remains of the right sinus venosus valve once it atrophies. The IVC will drain into the left atrium if the right sinus venosus valve does not regress and connect with the superior section of the septum secundum [14]. These two cases that we presented here demonstrate the diagnosis and treatment of two patients with abnormal IVC drainage to LA in a case of ASD. Our patients presented with normal saturations unlike the typical presentation of a patient with IVC to LA drainage. The reason could be the preferential streaming of the IVC blood into the RA which minimizes the right-to-left shunt. Computerized tomography (CT) angiography is useful to make a diagnosis [14]. But in our cases, as the diagnosis was made by echocardiography we went ahead with the surgical repair without a CT.

## Conclusions

We present two rare cases of IVC draining into LA presenting with normal saturation. Normal saturation thus doesn’t rule out anomalous systemic venous drainage. CT angiography is useful but not necessary to make a diagnosis. Surgery can be planned if the echocardiography is convincing enough. Utmost care should be taken while closing an OSASD and anomalous opening of the IVC into the LA should be ruled out in every case by identifying the opening of the IVC into the RA.
